# Quality as a drive-up digital teaching: Analysis of virtual classes in Colombian business schools

**DOI:** 10.1016/j.heliyon.2022.e09774

**Published:** 2022-06-22

**Authors:** Jose Andres Areiza-Padilla, Tatiana Galindo-Becerra

**Affiliations:** Department of Business Administration, Pontificia Universidad Javeriana, Bogotá 110231, Colombia

**Keywords:** Virtual classes, Quality, Business school, Digital teaching, University brand

## Abstract

The quarantines caused by the Covid-19, generated that thousands of university students had to abruptly abandon their classroom classes, and receive them in a virtual way. This sudden change caused a break in the daily life of thousands of university students, who were forced to use a teaching method that, although not new, was unusual at that time. This, initially generated a situation of rejection, stress and a perception of low quality classes that were received in a virtual way. However, with the passage of time, there was a need to know the perception of students about the quality of their virtual classes, but once the mandatory quarantine ended, because the literature has been focused only during the quarantine period. On the other hand, most studies on this topic have focused on developed countries, so there is little literature on the perception of virtual classes, taken by students from South America, whose countries have economic, social and technological characteristics, very different from developed countries. Based on the above, this study analyzes the perceptions of 867 students from 12 private business schools in the city of Bogota, Colombia. The data processed through PLS, allows us to observe that students have changed their negative perception towards virtual classes, and that, at present, these classes are considered of quality. Similarly, we can see that satisfaction, the willingness to continue using this methodology in the future, and the good performance of the university brand, are direct results of the quality of virtual classes.

## 1 Introduction

With the emergence of the Covid-19, all universities were forced to adopt online classes as a contingency to cope with the pandemic. Although virtual classes is not a new teaching methodology, its application was very limited, compared to the face-to-face classes until before the pandemic started. For more than 30 years, universities began to implement various educational formats, supported by technologies that improve their academic processes. However, completely online courses were very rare and in very low demand compared to face-to-face courses (Ebner et al., 2020).

This situation changed completely with the appearance of covid-19, since the pandemic made this type of education through virtuality the main teaching methodology, between the years 2020 and 2021. In most cases, university students had no choice but to receive classes online due to the quarantines imposed by the different governments, as a measure of anticipation against the pandemic.

According to Gonzalez et al. (2020), there are many differences on the impact that the pandemic had on the educational systems of the world, due to several factors that do not allow this comparison to be homogeneous. For example, each country has its own academic calendar, so academic activities started and are completed in different months of the year. For this reason, the holiday dates are also different between countries.

On the other hand, at the beginning of the pandemic each government made different recommendations to educational centers, for this reason while in some universities the vacations period was anticipated, in others the alternation between face-to-face and virtual classes was promoted, while others opted for completely virtual classes; however, in the end, all universities adopted virtuality during quarantines.

This unprecedented event in the world, generated that the entire academic community had to adapt to this new reality, in this way, the universities made great efforts to be able to train their teachers and their students in learning tools that applied this methodology (Soni, 2020).

Although at the beginning of the quarantine it was not possible to measure the impact of virtual classes on the teaching processes in higher education, currently some studies can be found in the context of the covid19, which allow us to observe how this abrupt change in the methodology of the classes, together with the compulsory isolation, provoked various negative feelings among the student population.

The first investigations on this subject show a widespread dissatisfaction and rejection by students towards virtual classes, in addition to their desire to return to face-to-face classes (Torres Martín et al., 2021). On the other hand, the studies of Garvey et al. (2021) tell us about the psychological impact that the strict and prolonged confinement of quarantines generate on students, explaining the emotional damage that these students experienced during the quarantine.

On the other hand, the studies of Pasion et al. (2021), allow us to see how the commitment, motivation and student's attachment to the university, were affected by the first outbreak of COVID-19.

However, other studies such as those by Gonzalez et al. (2020), show how e-learning with its various methodologies can improve the processes of autonomous learning by students, generating even higher academic performance. This was because during the quarantines by COVID-19, students were locked down in their homes and with more time available, for this reason, some of these students used this free time in a greater dedication in their own academic activities, and thus were more active and responsible with their learning processes.

However, these studies have focused on developed countries, where, due to the political, economic, technological and social characteristics of these countries, virtual classes before covid-19 already had a much higher level of penetration, compared to developing countries (Ssekakubo et al., 2011). In this way, their results cannot be generalized to countries with lower incomes because the implementation of virtual classes has different characteristics in this type of developing countries.

With this in mind, there is a need to know the perception of students, but from business schools located in South America, and how these students have faced the shift from face-to-face teaching to virtuality. For this reason, this research presents these two specific contributions.

First, this study seeks to understand the perception of university students about the quality of classes, which continue to be taught in a virtual way, once the mandatory quarantines have been completed. At the time of this study, it has been shown that many business schools continue to teach several online classes, although it is already possible to teach face-to-face classes, for this reason, virtual classes are no longer a completely unknown model for students, compared to the onset of the pandemic.

In this way, students have been using this methodology for several months and have had more time to learn about the benefits and disadvantages of this learning method. For this reason, their perceptions about the quality of the virtual classes, could have changed when comparing the beginning of the quarantine, with the completion of the same, due to the greater time they had to get to know this learning method.

Secondly, this study focuses on Latin America, more exactly in Colombia and makes it possible to contribute to literature from the perspective of developing countries, which, due to their technological and economic constraints, have had a learning process in a more limited way compared to developed countries.

In the particular case of Colombia, the previous studies of Martínez and Jiménez (2020) and Guzmán Rincón (2021) allow us to observe how this country presented several difficulties in the implementation of virtual classes and in general of the pedagogical model of e-learning. For this reason, there were high dropout rates as well as limited coverage under this type of pedagogical model, due to the economic limitations and technologies of that country. That is, it was a system with many limitations, and also with a low projection of growth and implementation in the near future.

It is important to mention that in Colombia on March 6, 2020, the first case of Covid-19 infection registered by the Ministry of Health was presented, taking into account this, the National Government formally decreed the start of the mandatory quarantine from 25 March 2020 to 13 April 2020.

In this way, business schools and in general all universities, had to abruptly start giving virtual classes in the face of the prohibition to teach face-to-face classes. These virtual classes were mostly conducted with modest preparation by teachers, most of whom had not done this type of teaching, and which was also considered momentary initially only for a couple of days of quarantine.

However, the national government extended the quarantine period in a way that an initial couple of days became several weeks and then several months. In Colombia, the quarantine was partially lifted on September 1, 2020, almost 6 months after its start date.

Due to the long period of quarantine, business schools and in general all universities had to carry out several training activities for their own teachers, where they were taught various strategies based on the e-learning methodology. Teachers were trained on how to use virtual platforms such as Teams, Google meet, Cisco Webex, Facebook Live, among others in order to generate a pleasant classroom experience.

However, great efforts were made to adjust teaching methodologies into an e-learning environment, so that professors did not just teach in a virtual mode, but to understand the different approach that this type of methodology needs, with a totally different pedagogy from the face-to-face environment.

Below are some of the activities that were developed to train professors in virtual classes: (1) Tips for keeping students connected, according to The Abdul Latif Jameel poverty action lab, (2) Webinar on virtual learning, (3) Technological tools for remote assessment, (4) Collaborative tools between students and teachers, (5) Design of evaluating rubrics.

In other words, the Covid-19 in Colombia forced Colombian teachers to understand the paradigm shift represented by this type of online methodology, from new forms of evaluation to new forms of interaction between students and teachers, to new ways of learning (Londoño-Velasco et al., 2021).

Based on the above, the structure of this research is as follows. First, an introduction is presented, then the theoretical framework of the variables is presented, finally the hypotheses and the model of this research are shared. For this research, the variables of: (1) student satisfaction, (2) attitude to continue using this methodology and (3) university brand performance, are considered consequences of e-service quality. The methodology developed is then explained, and the results of the data collected are then presented. Finally, the conclusions, limitations and future lines of research are presented, which can help to continue contributing to this topic.

## 2 Theoretical background and hypotheses development

### 2.1 Virtual classes quality

According to Saxena et al. (2021), the quality of virtual classes can be explained through three theories: The cognitive theory of multimedia learning, the social cognitive theory and the theory of the continuity model of information systems.

The cognitive theory of multimedia learning considers that students learn more effectively, through a combination of images and words and even audio, and not only with written text in the different pedagogical processes. In this way, in online education both the viewing of the website or online platform where classes are taught, along with an easy navigation of the multimedia application, are differentiating factors in improving the quality of online education service.

On the other hand, social cognitive theory indicates that one of the determinants of the satisfaction of virtual classes is the possible interaction between cognitive factors and environmental factors that affect student's behavior. Cognitive factors refer to expectations about the student's performance, while environmental factors refer to the social and physical environments that can affect the student's behavior in the online classroom. Finally, the theory of the continuity model of information systems refers to the possibility of the continuous use of a virtual classes platform and the intention of the student to continue using it (Saxena et al., 2021).

On the other hand, for Peltier et al. (2007), the quality of online teaching can be identified through the combination of six dimensions that are developed in the virtual classroom. The first one, regarding the interactions between students in class. The second one, regarding the interactions between the student and the professor. The third one, regarding the professor as supervisor and mentor of the virtual learning environment. The fourth one, refers to the quality of the classes. The fifth one, to the content of the course and the sixth one to the structure and organization of the online course.

Taking into account the university context, this study is based on the previous studies of Sultan and Wong (2019), which take the concept of the quality of services perceived by university students. In this way, these students, regarding the quality in the educational service they receive, define the perceived quality of service in students as a perceptive process of quality judgment.

This includes four assessments: an assessment in perception, an assessment in learning, an assessment in reasoning and an assessment of the understanding of the characteristics of the service; in addition it consists of three main dimensions: an academic dimension, an administrative dimension and a dimension related to the facilities where the service is provided (Sultan and Wong, 2019).

### 2.2 Student satisfaction regarding virtual classes

Athiyaman (1997) defines student satisfaction as a short-term attitude in which the student's educational experience is evaluated positively. In this way, universities promote student's satisfaction through various factors such as the declaration of their institutional mission, their goals, the institutions' objectives, and their educational project. All these generates a motivation in their students, which translates into high retention of current students and recruitment of new ones (Elliott, 2002).

Marsh and Roche (1997) developed a model that define students' satisfaction, taking into account several factors such as learning value. This model is based on the professor's attitudes related to their enthusiasm, sympathy, how organized he/she is and the interaction between them and the students. In other words, a high level of satisfaction in a class depends on the way the professor teaches.

With regard to online courses, Bangert (2006) identified four factors related to student satisfaction in this virtual modality, which include two-way interaction and communication between students and their teachers; the amount and quality of time in class, and the learning activities that are done with other classmates.

On the other hand, previous studies by Gray and DiLoreto (2016), indicated that the interaction among students in online classes, does not have a statistically significant impact on their level of satisfaction; however, the presence of the instructor highly impact the learning process in a positive way. In this way, it can be determined that students usually prefer a methodology where their online classes have some kind of contact and interaction with teachers through synchronous encounters, on those asynchronous classes where students have no interaction with the teacher, and everything is mediated through a platform.

In this way, the physical or virtual presence of the professor, is a significant factor for the student's perceived learning process. Similarly, how standardized is the structure of the online course is very important. In this way, the synchronous classes with physical interaction, organized structure and easy-to-follow course, are factors that increase satisfaction in students.

According to Pham et al. (2019), in the virtual classes’ environment, university students are consider by their schools as their clients. For this reason, one of the main objectives should be to generate satisfaction towards them through high quality online education. This virtual classes' quality can be achieved through various attributes, which make contribute to the overall quality of virtual classes, and in turn, positively affects student satisfaction.

For this research we assume that business schools in Colombia have managed to generate virtual classes that are perceived as high quality by students, thus generating greater satisfaction in those same students who use this method of learning. Therefore, and based on previous studies of Negricea et al. (2014); Pham et al. (2019); the following hypothesis is formulated:

Hypothesis 1 **(H1):** Virtual classes quality positively influences student satisfaction regarding virtual classes.

Although virtual classes already existed before covid-19, the pandemic forced universities that had face-to-face classes, to use this pedagogical strategy as a contingency to continue their academic process. Taking into account the previous studies of Almaiah et al. (2020); Maatuk et al. (2021); after several months receiving online lessons and once the mandatory quarantine was over, it is considered that students have assessed the positive aspects of this type of learning. This technology allow them to receive classes from anywhere in the world through different tools, without having to attend the campus in person.

For this reason, when students are satisfied about their virtual classes; they are more willing to continue using this methodology as a learning tool in the future. Taking into account the above, and as in the studies of Kenney and Khanfar (2009) ; Wen et al. (2011); it is considered that the students' satisfaction regarding virtual classes, will give these students the desire to receive again this type of classes. In the specific case of private universities, students will be willing to pay again and to receive classes through this methodology. With this in mind, the following hypothesis is formulated:

Hypothesis 2 **(H2):** Student satisfaction regarding virtual classes positively influences intent to continue using virtual classes in the future.

### 2.3 Intent to continue using virtual classes

A satisfied customer will have the intension to buy again in the same place they bought previously and where a positive shopping experience occurred. For this reason, in the same way that face-to-face sales offer a good shopping experience, electronic channels will generate customer satisfaction and the intention to buy again on that same online site (Wen et al., 2011).

However, for Chang et al. (2020); there is limited literature about the relationship between the intention to receive for the first-time online classes and the intention to receive online classes through the virtual classes’ methodology after using this educational service in the past. For this reason, it can be inferred that the intention to continue receiving online classes is positively correlated with the intention to pay for these classes. This means, there is a direct relationship between receiving online classes and the intention to pay for this educational service. In this way, when students receive online classes that they consider to be of high quality, they will be willing to continue paying for this type of educational service, but, if the student consider that the education they receive is not of high quality, he will not be willing to continue buying and using this type of technology for academic purposes.

In the educational field, according to Lin et al. (2011); students consider that virtual classes methodologies are useful when the learning process allows them to achieve their own personal goals, that where based on their educational purpose.

For Lin (2011); previous studies identified some factors that have a direct or indirect effect in the user's intentions of virtual classes’ continuity such as: user satisfaction, attitude, perceived utility, perceived ease of use and quality. However, the author considers that other aspects need to be considered, especially those related to quality, such as: (1) the quality of the service, (2) the quality of the system, (3) the quality of instruction and (4) the quality of the interaction.

The quality of the service (1) measures the difference between the user's expectations before taking classes and their final satisfaction level after attending class. (2) The quality of the system refers to the student's perception of how ease to use is the platform, as well as its reliability, speed, and the level of technical support available, in case they need this operational service. (3) The quality of instruction refers to the student's assessment regarding the content, the curriculum planning, the course design, didactic materials, and its comparison worldwide with similar classes. (4) Finally, the quality of interaction refers to the user's interaction perception of virtual classes (Lin, 2011).

Since virtual classes is developed through various electronic channels, for this investigation the intention to buy again refers to the intention of a client to continue receiving classes online in the future, based on their own previous consumption experiences.

In this way, and taking into account the studies of Lin et al. (2011); Lin (2011), it is considered that high virtual classes’ quality, generates in students the desire to receive again this type of classes through virtual methodologies, because of the positive experiences they had previously in which they achieved their goals. For this reason, we formulate the following hypothesis:

Hypothesis 3 **(H3):** Virtual classes' quality positively influences intent to continue using virtual classes in the future.

### 2.4 University brand performance

Universities are social organizations whose objective is to help human development and the social welfare of the community, for this reason, it is not appropriate to compare the concept of commercial brand performance with this type of organisations.

Brand performance in commercial terms refers to a successful brand which offers a competitive advantage. This makes the brand sustainable over time and generates a very positive economic return. Brand Performance in the terms of universities refers to the quality of the training provided by these educational centers, which is reflected in the academic quality of its students, and that also generates an important position within the academic rankings (Chapleo, 2010).

Nguyen et al. (2016) considers that, in the higher education sector, a student will generally evaluate the university's brand through a hierarchical sequence. In this way, the rational values and on a higher level, the emotional values, are involved. Brand performance will then be the result of that university's rational and emotional values through (1) brand image, (2) brand reputation and (3) brand commitment.

Based on Areiza-Padilla et al. (2021), it can be stated that for the education sector, the brand image refers to the identification of a university's brand through its symbols, which allow it to differentiate and position itself from other universities in an emotional and rational way. Brand reputation is a value judgment towards the university across time. This means, a favorable brand image that lasts longer (Nguyen et al., 2016).

The brand commitment from the student's perspective, is a desire to maintain a long-term relationship with the University. Such commitment reflects a student's motivation towards the institution and that maintains even after graduation (Nguyen et al., 2016).

According to Sultan and Yin Wong (2014), university brand performance is the perception that students have of the university's brand in the educational market. This is visible through: (1) the employment rate of graduates, (2) the starting salary after graduation, (3) the preference of companies to hire graduates from that university, (4) the pride and merit of the students for having a degree from that university, (5) the university's reputation (6) and the university's international position in terms of reliability.

In this way, the student's confidence towards their university's brand performance and the impact of this on their professional life after graduation, help the university as a marketing tool that promotes its educational offer through the academic programs offered in the market ().

Considering the above, for this research it is considered that a high perception of virtual classes’ quality from students will help to increase the University Brand Performance. In the context of Covid-19, all university students received this type of educational training on a compulsory basis. This generated an approach of thousands of students to this type of online teaching, which had a very small impact before the pandemic, compared to face-to-face teaching.

Although at the beginning of the pandemic, virtual classes where used only as a contingency, nowadays universities can find in this methodology a tool that helps them to improve the brand performance and to adapt to a new post-pandemic reality where online teaching has a greater relevance.

**Figure 1:**
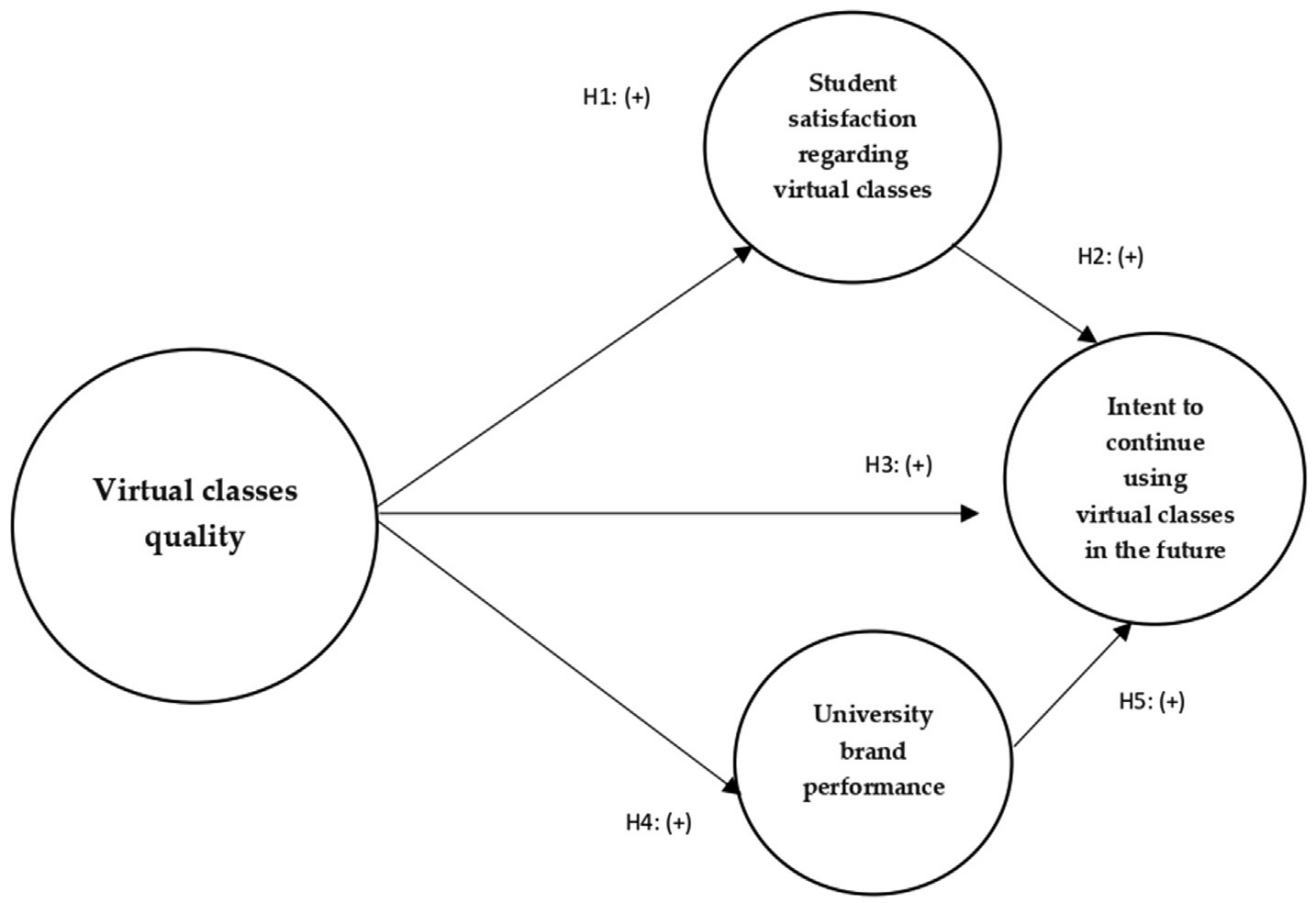
Research model. Source: Author's own compilation.

In this way, having higher quality standards in online education will positively affect the overall performance of the university's brand image. This in turn will generate a greater reputation of the brand, so students will be willing to continue to attend online classes from this university in the future. Taking into account the studies of Sultan and Yin Wong (2014, 2019), Chaudhary et al. (2020) the following hypothesis is formulated (see Figure 1):

Hypothesis 4 **(H4):** Virtual classes quality positively influences university brand performance

Hypothesis 5 **(H5):** University brand performance positively influences intent to continue using virtual classes in the future.

## 3 Materials

### 3.1 Participants and procedure

The data collection process was carried out through structured questionnaires through the Qualtrics software and sent over the Internet. A non-probabilistic sampling approach was used for convenience due to limited resources, information, and the need to collect information quickly. The questionnaire was sent exclusively aimed at students from private business schools in Bogota, Colombia (South America) that had the recognition of accreditation of high quality, issued by the Ministry of Education of this country. For this, the students had to indicate at the beginning of the questionnaire, to which business school they belonged, and in case their school was not in the form, the system did not allow them to continue to carry out the other questions.

A snowball sampling method was performed, where the participants could share the questionnaire with other participants. For this, each researcher sent the questionnaire to their respective groups in the different social networks, where members of the group were invited to share the questionnaire with other interested people.

The process for sample collection, data processing and informed consent follows ethical guidelines reviewed and approved by the ethics committee of the Javeriana University with code FCEA-DF-0092-2021.

At the beginning of the questionnaire, participants were informed that this activity was voluntary and without any financial remuneration; and that the results would be for academic purposes. Likewise, they were told that the data collected would be treated in a global way and not in a particular way; for this reason, no personal data were required. Participants were free not to continue to complete the questionnaire at any time.

The sample was based on university students over 18 years of age, who were of Colombian origin, or foreign students who were from South America and who were students in Colombia.

To carry out the survey it was necessary that the participating students had attended or were attending classes in the virtual mode, with synchronous events between teacher and student during the first and/or second semester of 2021. That is, once the mandatory quarantine ended, since it was sought to know their perception only about the classes that continued using this methodology, although it was already allowed to have face-to-face classes.

In Colombia, the first academic semester goes from January to June and the second period from July to December. The data obtained were collected between the first and second half of 2021.

Students had to be from any business school in Bogota, Colombia; but these schools should be exclusively private, that is to say, students from public business schools were not taken into account. For this, one of the initial and excluding questions of the form was the public or private character of the student's business school.

Taking this into account, we proceeded to group the Careers by name similarity, in this way the students participating in the survey had to choose one of the following options, which are the most demanded careers in Colombia in the business area: Business, tourism, administration, international trade, sales, finance, advertising, marketing, accounting, or other business-related careers, not explicitly mentioned in previous careers.

Taking this into account, 867 valid questionnaires were obtained from 12 private schools in Bogota which have high quality accreditation. Bogota has a total of thirty universities, of which six are public and twenty-four are private. Of the thirty universities, only twenty-one have high quality accreditation, issued by the National Accreditation Council of the Ministry of Education, of which four are official and seventeen are private. These data were processed through PLS, which allows working with small sample sizes Peng & Lai (2012), Memon et al. (2020).

Of this sample, 53.9%were women and 46.1%were men. Regarding age, 63.6% were between 18 and 25 years old, 34.3% were between 26 and 35 years old and only 2.2% over 36 years old. Table 1 shows the sample distribution.

**Table 1:** Sample distribution.

	N	%
**Gender**		
Men	400	46,1%
Women	467	53,9%
**Age**		
18–25	551	63,6%
26–35	297	34,3%
36–45	19	2,2%
**Career**		
Business/Administration	317	36,6%
Tourism	78	9,0%
International trade	45	5,2%
Marketing/Sales/Advertising	263	30,3%
Finance/Accounting	140	16,1%
Other related.	24	2,8%
**Nationality**		
Colombia	719	82,9%
Foreign students in Colombia (From South America)	148	17,1%

Source: Author's own compilation.

### 3.2 Measures

A 7-point Likert scale was used to record the responses, where 1 was "totally disagreeing" and 7 ″totally agreed". For this specific research we decided to use a 7-point Likert scale, because studies by Diefenbach et al. (1993) found that 7-point Likert scales tend to be more sensitive than the 5-point scale.

The scales were extracted from the academic literature, and translated into the Spanish language, preserving the grammatical meaning of the questions and adapting them to this research.

To measure the variables of "Virtual classes’ quality" and "Student satisfaction regarding virtual classes", the scales of Udo et al. (2011) with four items for each variable were used. To measure the variable of "Intent to continue using virtual classes " this study used three items from Chang et al. (2020); with two items of Udo et al. (2011) since they were better explained this way. Finally, to measure the variable "University brand performance", the eight items of the Sultan and Wong (2019) scale was used. The description of the scales used is detailed below in Table 2:

**Table 2:** Sources of measured items.

Study variable	Author(s)	Item(s)
Virtual classes quality	Udo et al. (2011).	4
Student satisfaction regarding virtual classes	Udo et al. (2011).	4
Intent to continue using virtual classes	Chang et al. (2020); Udo et al. (2011).	5
University brand performance	Sultan and Wong (2019).	8

Source: Author's own compilation.

## 4 Results

The data was processed through the PLS-SEM 3.2.7 program. First, the validation of the measuring instrument was carried out, taking into account the evaluation of the validity and reliability of the model.

The validation of the measuring instrument was performed by analyzing the reliability of the variables, for which the individual reliability (Cronbach α) and also the measurement of composite reliability (CR) with values greater than 0.7 were considered. Convergent validity was used for the Average Variance Extracted (AVE). The results show that all the loads of the variables were significant and higher than 0.7. On the other hand, the Average Variance Extracted (AVE) value of each variable is greater than 0.5. These data demonstrate an adequate convergent validity in the measurement model of Fornell and Larcker (1981). In this way, the reliability and convergent validity of the scales is confirmed which measures the four variables presented in the research model.

After this, the structural model was estimated, evaluating the weight and magnitude of the relationships between the different variables.

Table 3 shows the descriptive results of the four variables in this study, with their respective items. It should be noted that all items have a value above four, on a scale of 1–7, where the highest value with 5.89 is the item of "I would likely do another degree program online" which corresponds to the variable of "Intent to continue using virtual classes ".

**Table 3:** Measurement model evaluation results.

Variables/Items	Mean	St. Dev	Loadings Factor
**F1. Virtual classes quality (α = 0.798; CR = 0.753; AVE = 0.711)**			
The web site for virtual classes seems to be up to date	4.33	1.78	0.837∗
The web site for virtual classes works well	4.28	1.74	0.787∗
The web site for virtual classes has clear instruction.	4.17	1.63	0.768∗
Your perception of the overall quality of the education you get through virtual classes is excellent	4.29	1.63	0.789∗
**F2. Student satisfaction regarding virtual classes (α= 0.709; CR= 0.730; AVE= 0.739)**			
I am satisfied with my decision to enroll in the online classes	5.18	1.88	0.723∗
My choice to enroll in online classes was a wise one	4.87	1.46	0.799∗
I think I did the right thing when I paid for online learning service	4.09	1.65	0.834∗
I feel that my experience with online learning has been enjoyable	5.22	1.74	0.778∗
**F3. Intent to continue using virtual classes in the future (α= 0.729; CR= 0.798; AVE= 0. 724)**			
If there is a need to learn in the future. I will use virtual classes to learn.	4.49	1.71	0.818∗
Besides using the university website to learn. I will also use other virtual classes tools more frequently and actively.	4.83	1.87	0.967∗
I will suggest others to use virtual classes to learn.	5.45	1.99	0.776∗
I know I get good service for the fee I paid for online distance learning	5.02	1.63	0.702∗
I would likely do another degree program online	5.89	1.84	0.943∗
**F4. University brand performance (α= 0.750; CR = 0.764; AVE= 0.887)**			
This university as a brand is reliable	4.76	1.92	0.878∗
A degree from this university is worthy	4.88	1.74	0.784∗
This university performs well	4.78	1.79	0.869∗
I found that this university has a good reputation	4.33	1.94	0.801∗
I am proud to be a student of this university	4.23	1.77	0.794∗
A degree from this university enhances employability	4.76	1.54	0.904∗
The graduates of this university receive a good salary	4.87	1.63	0.731∗
Employers prefer graduates from this university	4.84	1.62	0.776∗

α = Cronbach's Alpha; CR = Composite reliability; AVE = Average Variance Extracted; ∗p < 0.01.

Source: Author's own compilation.

At a general level, the results show that virtual classes analyzed through the four variables of this study, had a very positive acceptance by Colombian business students.

According to Fornell and Larcker (1981), the discriminating validity must take into account the square root of the AVE of each variable. In this way this result must be greater than the correlations it has with the rest of the variables in the model. Table 4 shows that all the square roots of the AVE of each variable are greater than the correlations with any other variable in the model. For the ratio (HTMT), 0,9 is considered as the appropriate maximum cut-off value. The results of this research in Table 2 show that all the values of the ratio (HTMT) are below 0.9.

**Table 4:** Analysis for discriminant validity.

	Virtual classes quality	Student satisfaction regarding virtual classes	Intent to continue using virtual classes in the future	University brand performance
Virtual classes quality	**0.823**	0.289	0.238	0.279
Student satisfaction regarding virtual classes	0.278	**0.798**	0.183	0.428
Intent to continue using virtual classes in the future	0.309	0.129	**0.720**	0.164
University brand performance	0.178	0.430	0.238	**0.874**

Note: On the diagonal: square root of the AVE values. Below the diagonal: correlations. Above the diagonal: HTMT values.

Source: Author's own compilation.

After this process, the PLS-SEM proceeded to estimate the structural model with 5000 subsamples using bootstrapping Henseler (2017). These results allow us to show that trajectory coefficients are significant in all cases and in the same sense that were formulated in the hypotheses. The coefficients of determination R^2^ and Q^2^, are higher than 0. This allows us to check the explanatory effect of the structural model through Table 5.

**Table 5:** Results of the structural equations model.

Hypothesis	Relationship	β	t	p-Value	Contrast
H1	F1 –F2 Virtual classes quality -Student satisfaction regarding virtual classes	0,343	3,679	0,015	Supported
H2	F2–F3 Student satisfaction regarding virtual classes -Intent to continue using e-learning in the future	0,265	3,125	0,000	Supported
H3	F1–F3 Virtual classes quality -Intent to continue using e-learning in the future	0,283	2,878	0,025	Supported
H4	F1–F4 Virtual classes quality -University brand performance	0,317	2,980	0,008	Supported
H5	F4–F5 University brand performance -Intent to continue using e-learning in the future	0,278	3,677	0,000	Supported

Note: R^2^ (F2) = 0.189; R^2^ (F3) = 0.256; R^2^ (F4) = 0.487; Q^2^ (F2) = 0.298; Q^2^ (F3) = 0.256; Q^2^ (F4) = 0.172.

Source: Author's own compilation.

In this way, it can be observed that all the relationships between the variables were positive and significant among them; allowing to confirm the five hypotheses that were proposed in this research.

## 5 Conclusions and discussion

### 5.1 Theoretical contribution

This research want to contribute to the scarce literature that exists on the perception that students have of business schools in South America, more exactly in Colombia; in the quality of their classes that continued to be taught in a virtual way, but after the end of the mandatory quarantine caused by the Covid-19.

In this way, the results of this research allow us to present two specific contributions. As a first development, this research has been able to show that virtual classes, although they were a convenient novelty at the beginning of quarantine, because students were forced to this type of teaching which was unusual at that time; Nowadays and with the passage of time, virtual classes are part of a new daily life for students, who have already become familiar with this type of virtual methodologies.

In this way, although several previous studies, such as those of Torres Martín et al. (2021). Garvey et al. (2021), Pasion et al. (2021), carried out at the beginning and during the pandemic, on the perceptions of the virtual classes and the different methodologies of the e-e-learning, showed how students could have certain feelings of rejection and frustration to this type of virtual teaching, with the results of this research it can be evidenced that that perception has changed, decreasing so much the rejection towards virtual classes, such as the perception of low quality virtual classes, purchased with face-to-face classes; even highlighting the benefits of this type of online teaching at the university level.

For this reason, for this research we considered that although virtual classes already existed before covid-19, their implementation was very limited in all academic areas. However, quarantines forced to adopt this methodology abruptly, that like any change, usually generates a lot of resistance at the beginning, even with time, we can say that e-learning and all its methodologies including virtual classes, have managed to make a niche in the university education of business schools in a satisfactory manner.

In other words, once quarantines have ended and students have continued using this methodology, they have had more time to learn the advantages and disadvantages of this learning method and highlighted the benefits that this type of virtual teaching brings.

On the other hand, the second novelty of this research has managed to contribute to the scarce literature that exists on virtual classes from the perspective of developing countries, especially in Latin America, and more precisely in Colombia. This is because most studies have focused on analyzing the teaching processes through e-learning in times of the Covid-19, but developed countries, which have greater technological advances and greater economic and human resources, compared to developing countries.

Taking this into account, this research has managed to determine that the quality of virtual classes manages to have a positive and significant impact on student satisfaction regarding virtual classes; allowing us to confirm hypothesis 1 (H1), as well as the previous studies of Negricea et al. (2014), Pham et al. (2019). In this way, it begins to show that such satisfaction on the part of students, although it used to be associated mostly with face-to-face classes, currently also associated with virtual classes, which are now not seen as a novelty, but as a new every day.

On the other hand, this research was able to confirm the positive and significant relationship that exists between student satisfaction regarding virtual classes and the intent to continue using virtual classes in the future, being able to verify hypothesis 2 (H2), like previous studies by Kenney and Khanfar (2009), Wen et al. (2011). For this reason, and based on the results obtained, for this research we consider that, although the virtual classes were a contingency response to the quarantine of Covid-19, at present they represent a new academic reality, which does not compete with the presence, but complements it.

In the same way, this research was able to confirm the positive and significant relationship between virtual classes quality and the intent to continue using virtual classes in the future, as well as previous studies of Lin et al. (2011), Lin (2011), succeeding in confirming hypothesis 3 (H3).

In this way, we can show how globalization not only impacts commercial transactions but also through technology since business schools can already reach anywhere in the world through their virtual classes without the need for pupils and teachers to leave their country of origin. This allows to further enrich the learning process between students and teachers with interactions from anywhere in the world with a global projection.

In other words, new digital technologies have made it possible to connect the Internet, resources and people around the world to further improve educational processes. In fact, the previous studies of Reddy et al. (2021) allow us to see how computer competence and computer self-efficacy have a positive and significant impact on the acceptance of technology, that is, they are significant predictors of students' intention to want to use technology in their learning processes.

On the other hand, it is also considered that the educational demand for this type of teaching methodology will increase over the years, especially for students from South America, where they will be able, for example, to receive classes from European business schools of North America and Asia, without having to leave their country of origin.

Finally, this research also analyzed the positive and significant relationships between the quality of virtual classes and the performance of the university brand in hypothesis 4 (H4) and the positive and significant relationship between the performance of the university brand and the intention to continue using virtual classes in the future of hypothesis 5 (H5). Thus, hypotheses 4 and 5 (H4–H5) were contrasted, as in previous studies by Sultan and Yin Wong (2014, 2019), Chaudhary et al. (2020).

Based on the above, this research found that the quality of virtual classes helps improve teaching processes, but also benefits the brand of the business school, which are offering classes with this methodology, after the end of compulsory quarantine. In this sense, business schools end up improving their own reputation, offering a teaching method that allows greater flexibility and that adapts to the world after the pandemic.

### 5.2 Managerial implications

The covid-19 completely changed many everyday aspects of people, modifying their habits, behaviors and routines, which had to adapt to a new reality in pandemic. The educational sector, and especially the business schools, were not oblivious to these changes, where they had to change their classroom classes in a mandatory and even improvised way, for virtual classes, generating many learning difficulties with this methodology at the beginning of quarantine. However, over time, these same difficulties have become opportunities to improve teaching processes through technology, opening the door to a new reality in the education sector. This e-learning tool, while not new, had not penetrated significantly into business schools until before Covid-19. However, once the transition to this methodology has been overcome during the quarantine period, a turning point has been observed in the academic community, which was able to observe and analyze the advantages of this type of methodology.

Over time, these same difficulties have become opportunities to improve teaching processes through technology, opening the door to a new reality in the education sector. This e-learning tool, while not new, had not penetrated significantly into business schools until before Covid-19. However, once the transition to this methodology has been overcome during the quarantine period, a turning point has been observed in the academic community, which was able to observe and analyze the advantages of this type of methodology.

In this way a new learning scenario has been generated, because based on the results obtained in this research, we consider that, despite the return to face classes, The virtual classes came to stay and offer a better learning experience for both the student and the teacher, combining the best of both methodologies, and allowing the student to combine presence with virtuality.

In this way, the results of this research show that, once the resistance to the unknown and the technical problems that arise in virtual classes are overcome, it can be demonstrated that the level of acceptance of students to virtual classes has increased, and especially to improve their perception of quality towards them. This situation at no time poses a threat to face classes, but, on the contrary, evidence as virtual classes are becoming a strategic ally for teaching processes in business schools.

### 5.3 Limitations and future research

Below are some limitations that this research had, which could also be considered as future lines of research. The first is the geographical scope of the sample, since it focuses only on students from Bogota, Colombia. It is important to replicate this study in other cities of the country, we even consider interesting to replicate this study in other similar economies in Latin America, in order to identify possible similarities or differences, taking into account cultural differences between countries.

On the other hand, this study focused on undergraduate students, for this reason the opinion of graduate students is unknown. It would be interesting to know the opinion of postgraduate students, since most of these students usually have previous work experience or simultaneous to their postgraduate studies, which could positively or negatively influence their perception of the quality of virtual classes. On the other hand, graduate students usually have less free time, since it is common for graduate students to receive classes at night, and work during the day, that is to say, unlike undergraduate students, graduate students usually do not have so much time for their classes or for their life on campus.

It is also important to emphasize that this study was based on private business schools, which is why it would be interesting to know the perception of students, but of public business schools, to make a comparison between the opinion of students in public and private schools, and to analyze whether free education or paid education, generates differences in the perception of virtual classes and the quality of these classes. In the same way, we believe that new research should be developed, in which this study can be replicated, but focused on other careers than business, such as medical careers, arts and engineering, which have very distichous characteristics, to the careers of social sciences.

On the other hand, and based on the studies of Areiza-Padilla et al. (2021) we consider it important to analyze the virtual classes offered by business schools, but not those of an academic nature, but those that are complementary in the education of the student, as sports or cultural, to know the perception of the students with these classes of non-professional character. Since it has been shown, as sports classes online, begin to become relevant in a post-pandemic world.

Finally, it is also important to be able to identify the background in the implementation of virtual classes in business schools, which ensure the success of this type of teaching through the quality of these classes. In this way, public and private business schools will be able to focus their efforts on improving their quality processes, in order to obtain better academic results from their students.

## Declarations

### Author contribution statement

All authors listed have significantly contributed to the development and the writing of this article.

### Funding statement

This research did not receive any specific grant from funding agencies in the public, commercial, or not-for-profit sectors.

### Data availability statement

Data included in article/supplementary material/referenced in article.

### Declaration of interests statement

The authors declare no conflict of interest.

### Additional information

No additional information is available for this paper.
